# Leveraging Monaural Exposures to Reveal Early Effects of Noise: Evidence from Police Radio Ear-Piece Use

**DOI:** 10.1177/23312165251410988

**Published:** 2026-01-30

**Authors:** Hannah Guest, Paul Elliott, Martie van Tongeren, Joseph Laycock, Steven Thorley-Lawson, Michael A. Stone, Michael T. Loughran, Christopher J. Plack

**Affiliations:** 1 Manchester Centre for Audiology and Deafness, School of Health Sciences, 5292 The University of Manchester, Manchester, UK; 2 MRC Centre for Environment and Health, Department of Epidemiology & Biostatistics, School of Public Health, 4615 Imperial College London, London, UK; 3 Division of Population Health, Health Services Research & Primary Care, School of Health Sciences, The University of Manchester, Manchester, UK; 4 272245 Ansys UK Limited, Sheffield, UK; 5 8228 West Yorkshire Police, Wakefield, UK; 6 Department of Psychology, 4396 Lancaster University, Lancaster, UK

**Keywords:** noise exposure, noise-induced hearing loss, tinnitus, temporary threshold shift, occupational health, police health

## Abstract

Research into the long-term effects of noise on hearing is often confounded by health and lifestyle differences between individuals. UK police radio ear-pieces are capable of emitting high sound levels and, crucially, are worn in one ear, allowing between-ear comparisons which control for individual-level confounding factors. Low volume-control settings are recommended to reduce risk to police hearing, yet actual usage patterns and auditory effects remain unexamined. This study used a large-scale survey (*N* = 4,498) to assess ear-piece noise exposure and the associated hearing health. Most participants reported using high volume-control settings and 45.2% reported experiencing signs of temporary threshold shift (TTS) in the exposed ear. Estimated weekly-averaged noise exposures frequently exceeded the UK's 85 dBA Upper Exposure Action Value. Ear-piece use was associated with 73% (95% confidence interval [CI] 46–106%) increased risk of persistent tinnitus, which on mediation analysis appeared to be driven by a subset of users who experienced signs of TTS. Importantly, tinnitus location was associated with the side of exposure, suggesting tinnitus related to device use rather than to other factors. In contrast, Digits-In-Noise thresholds showed no relation with noise exposure; potential explanations include compensatory auditory training effects, but limitations of Digits-In-Noise data must also be considered. Findings highlight a need for further investigation into hearing risks in police personnel, including in-person auditory testing. Risk mitigation strategies might involve improved device design, training on safe use, and expanded hearing health surveillance. Given the potential for cumulative auditory damage, TTS may serve as an early warning sign, warranting attention in broader noise-exposed populations.

## Introduction

Hearing loss is the third-leading cause of years lived with disability (World Health Organization, 2021) and noise exposure is the primary cause of preventable hearing loss (Rabinowitz, 2000). Noise-induced hearing loss (NIHL) is a significant public health concern, with substantial implications for communication and quality of life. Typically, noise exposure affects both ears, in both occupational and recreational settings. Monaural or asymmetric exposures are less common, often arising from specialized circumstances such as firearm use (Cox & Ford, 1995), and present a powerful research opportunity. Within-subject comparisons between the noise-exposed and control ears reduce interindividual variability and confounding factors, a widespread issue in auditory research (Bharadwaj et al., 2019; Plack et al., 2016). This methodological advantage is particularly relevant for identifying early-stage NIHL, which might otherwise be masked by unrelated between-subject differences in auditory function.

Auditory research increasingly prioritizes the early identification of noise-induced hearing damage, including “subclinical” auditory pathologies. Traditionally, NIHL research has depended largely on a single diagnostic tool: pure-tone audiometry (PTA), which measures the softest sounds a person can hear. Yet early noise-induced damage may be subclinical, without measurable effects on PTA (Plack et al., 2014; Le Prell et al., 2023). Following the seminal findings of Kujawa and Liberman (2009), an explosion of research has focused on cochlear synaptopathy—damage to the synapses between hair cells and auditory nerve fibers (Bramhall et al., 2019; Plack et al., 2016). A wider array of pathophysiologies is now thought to contribute (Valderrama et al., 2022), including subclinical damage to cochlear outer hair cells (Poling et al., 2022) and extended-high-frequency hearing loss (Lough & Plack, 2022). Considering the early effects of noise more broadly, the “normal” range of PTA thresholds is wide, spanning 30 dB. Taking a broader view still, mild and unilateral hearing loss often remains undiagnosed in the population. While the long-term effects of chronic noise exposure are well documented, the early warning signs remain less understood, raising the possibility that noise-induced damage is more widespread than previously estimated.

Possible perceptual consequences of early noise-induced hearing pathology are of particular interest, not only due to their impacts on quality of life but also their diagnostic potential. Links between cochlear synaptopathy and tinnitus have been investigated, but findings to date are mixed (e.g., Guest et al., 2017; Schaette & McAlpine, 2011). Subclinical deficits have also been examined in relation to speech perception (e.g., Guest et al., 2018; Holmes & Griffiths, 2019). However, substantial effects of synaptopathy on speech-in-noise performance may be unlikely (Oxenham, 2016), especially when investigated using conventional speech-in-noise tasks not optimized to detect this pathology (DiNino et al., 2022). The early effects of noise extend beyond synaptopathy, and monaural exposure effects may be especially detectable using tasks that exploit binaural cues or that directly compare between-ear performance. Finally, there is growing recognition that “temporary” threshold shifts can mask permanent auditory injury (Kujawa & Liberman, 2009), and that associated short-term perceptual changes might serve as indicators of cumulative damage (Brungart et al., 2025).

Beyond the context of subclinical pathologies, the early indicators of NIHL may be valuable where individuals are exposed to noise but not routinely monitored via PTA. Examples include concertgoers and nightclub attendees, who lack the regulatory protections afforded to workers in similarly noisy environments (Health & Safety Executive, 2005) due to the voluntary nature of their exposure. Other examples include workers exposed to noise sources whose risks to hearing are not yet adequately understood or controlled. As awareness grows about the broader consequences of noise exposure, leveraging robust research designs to identify early markers of auditory damage can potentially inform risk-reduction strategies and hearing-health surveillance.

Monaural noise exposures from headsets and ear-pieces are common in several occupational settings, such as call centers, retail environments, and emergency services. Importantly, they provide a more representative model of continuous, asymmetric noise exposure than impulsive sources like firearm noise. Over the past two decades, UK police personnel have used TETRA (Terrestrial Trunked Radio) personal radios. Officers generally listen through an ear-piece worn in a single ear (Elliott et al., 2014). Device models vary between police forces, but are typically capable of emitting high sound pressure levels, about 100 dBA (ISVR Consulting, 2010). Reports on the devices have emphasized that users must select low volume-control settings to minimize the risk of auditory damage (Health & Safety Laboratory, 2004; ISVR Consulting, 2010). To date, no research has investigated the volume-control settings selected by police personnel, nor the impacts on their auditory health. This presents a critical research opportunity, not only to inform strategies for preserving police hearing health, but also to enhance understanding of the early indicators of noise-induced hearing deficits.

### Study Aims

The study aimed to describe ear-piece exposures and infer their consequences for the hearing health of police personnel. A detailed questionnaire and online listening task were designed to allow data collection from a large sample, including between-ear comparisons. The study was designed to address the following primary research questions (RQs), of which the first two are descriptive and the remainder inferential:

- What is the distribution of ear-piece noise-exposure levels among ear-piece users? (RQ1)
- How often do ear-piece users experience signs of temporary threshold shift (TTS; temporary tinnitus and/or muffled hearing) in the exposed ear after wearing an ear-piece? (RQ2)
- Do signs of ear-piece-associated TTS occur more frequently in users who select higher volume-control settings? (RQ3)
- Is ear-piece use associated with an increased risk of prolonged spontaneous tinnitus? (RQ4)
- Are signs of ear-piece-associated TTS associated with an increased risk of prolonged spontaneous tinnitus? (RQ5)
- Is tinnitus location related to the side on which the ear-piece was worn? (RQ6)
- Is ear-piece use associated with poorer Digits-In-Noise (DIN) perception in the exposed ear than in the control ear? (RQ7)

To harness the benefits of between-ear comparisons, RQ4 and RQ6 are designed to be interpreted in tandem. RQ4 examines whether an association exists between ear-piece use and tinnitus that warrants further explanation, while RQ6 evaluates whether ear-piece use itself may contribute to this association, benefiting from internal control that minimizes nearly all exogenous and endogenous confounding factors. Similarly, RQ7 employs a within-subject design to control robustly for confounding influences.

In addition, following the registered protocol, a number of secondary RQs were addressed (results are reported in the Supplemental materials):

- DIN thresholds: (a) Is ear-piece use associated with poorer “antiphasic” DIN thresholds (a form of DIN testing reliant on binaural processing)? (b) Is total energy of ear-piece noise exposure associated with poorer DIN thresholds in the exposed ear than in the control ear? (c) Is total energy of ear-piece noise exposure associated with poorer antiphasic DIN thresholds? (d) Are signs of ear-piece-associated TTS associated with poorer DIN thresholds in the exposed ear than in the control ear? (e) Are signs of ear-piece-associated TTS associated with poorer antiphasic DIN thresholds?
- Self-reported diagnosed hearing loss: (f) Is ear-piece use associated with an increased risk of diagnosed hearing loss? (g) Are signs of ear-piece-associated TTS associated with an increased risk of diagnosed hearing loss? (h) Is the laterality of diagnosed hearing loss related to the side on which the ear-piece was worn?
- Tinnitus: (i) Is the total energy of ear-piece noise exposure associated with an increased risk of prolonged spontaneous tinnitus?
- Demographic factors: (j) Does age influence auditory outcomes? (k) Does sex influence auditory outcomes?

## Method

### Registered Protocol

A comprehensive study protocol was registered via the Open Science Framework (https://osf.io/4v59u/files). All data were collected, processed, and analyzed as specified in the protocol. At the time of registration in June 2024, preliminary data had been collected from 702 participants (∼15% of the full sample), in the course of an internal pilot phase. Two corrections were made to the protocol in March 2025, one refining the values used to convert volume-control settings to estimated sound levels, the other correcting a misnamed variable (highlighted in the updated protocol). In the present study, study methods are described in brief; readers may consult the registered protocol for full details.^1^

### Participants

The Airwave Health Monitoring Study of the British police forces was established in 2004 to investigate possible long-term health effects associated with use of the TETRA personal radio system (Elliott et al., 2014). Of the ∼50,000 Airwave participants, approximately 28,000 were contacted by email inviting them to take part in the present study. Respondents were 4,890 (17.5%) current and past police personnel, each of whom provided written informed consent for collection of hearing data. Respondents were excluded if they reported having hearing loss that developed before first wearing an ear-piece and/or hearing loss due to ear disease, illness, injury, medication, or surgery (*n* = 339). Hearing losses with more routine causes, such as aging and noise exposure, were not criteria for exclusion. The protocol also allowed for exclusions on the basis of data quality. Accordingly, data from a further 53 respondents were excluded due to inconsistencies in the survey data (e.g., stated number of years of ear-piece exposure exceeded 25). The remaining sample comprised 4,498 (92.0%) participants, exceeding the target sample size of 4,000 indicated by statistical power analysis. Table 1 lists demographic characteristics of the excluded respondents and the remaining evaluable sample.

**Table 1.** Demographic and Hearing Characteristics.

	Excluded Respondents	Evaluable Sample
*N*	392	4,498
Sex	278 (71%) male; 114 (29%) female	2,975 (66%) male; 1,523 (34%) female
Age	Mean = 55.9 (SD = 8.2) years	Mean = 55.6 (SD = 8.6) years
	Median = 56 (IQR = 10.0) years	Median = 56 (IQR = 12.8) years
Ethnicity	299 (97%) White; 0 (0%) Black; 1 (0%) Asian; 1 (0%) mixed; 6 (2%) other; 85 not stated	3,487 (95%) White; 28 (1%) Black; 55 (2%) Asian; 36 (1%) mixed; 49 (1%) other; 843 not stated
Ear-piece use	155 (40%) nonusers; 190 (48%) unilateral users; 47 (12%) nonunilateral users	2,164 (48%) nonusers; 1,841 (41%) unilateral users; 493 (11%) nonunilateral users
Presence of prolonged spontaneous tinnitus	238 (61%) no; 154 (39%) yes	3,735 (83%) no; 763 (17%) yes
Location of prolonged spontaneous tinnitus	77 (50%) central; 38 (25%) unilateral; 39 (25%) bilateral but asymmetric	458 (60%) central; 121 (16%) unilateral; 184 (24%) bilateral but asymmetric
Presence of diagnosed hearing loss	209 (53%) no; 183 (47%) yes	4,037 (90%) no; 461 (10%) yes

*Note*. IQR = interquartile range.

### Study Design

Data collection commenced with an online questionnaire (see https://osf.io/4v59u/files for question wording). Following the provision of informed consent and determination of eligibility, the remaining questions assessed ear-piece exposure and auditory health. At the close of the questionnaire, the participant was presented with an individualized link to a speech-perception task, which they could complete immediately or access later if more convenient.

All study procedures were approved by the NHS North West (Haydock) Research Ethics Committee (reference no. 23/NW/0350). Since the study recruited from the Airwave Health Monitoring Study cohort, it was additionally reviewed and approved by the Airwave Data Access Committee (reference no. AH-EXT-147).

### Ear-Piece Exposure

Participants were asked if they had ever regularly worn a TETRA ear-piece. Ear-piece users were asked to report which ear(s) the ear-piece was worn in and the reason for their choice of ear(s). Reporting methods developed in collaboration with current police personnel were then used to obtain data on durations and levels of ear-piece exposure over the career. A tabular structure within questionnaire software allowed them to divide their career into a number of discrete periods, each of which could have different ear-piece usage patterns. This division allowed for changing exposure habits as staff moved between job roles. Since policing environments vary in environmental noise levels, participants were asked to dichotomize their exposure habits into times when a “loud” volume-control setting was used (typically in noisy environments) and times when a “normal day-to-day” volume-control setting was used (typically in quieter environments). Importantly, these plain-language loudness descriptors were not used directly to estimate exposure levels. Instead, participants estimated the approximate volume-control dial position that corresponded to each of these two settings, expressed as a percentage of maximum volume. Finally, for each career period, they were asked to report (a) the duration of the period in years, (b) the number of hours per week the ear-piece was used, and (c) the proportion of that time during which they used the “loud” volume-control setting specified above.

### Auditory Health

#### Tinnitus

Tinnitus definition can radically alter reported prevalence (McCormack et al., 2016). Care was taken to restrict the study's main tinnitus metric to “prolonged spontaneous tinnitus”: tinnitus that occurred spontaneously (not only after noise exposure) in the past 12 months and lasted for >5 min at a time. This definition excludes transient tinnitus (which is common and less likely to reflect auditory pathology) and TTS-related tinnitus (which was assessed separately; see “Signs of Ear-Piece-Associated TTS” section below). Consistent with these distinctions, the prevalence of *prolonged spontaneous* tinnitus (17%) was around half that of spontaneous tinnitus *of any duration* (30%), which in turn was lower than the prevalence of tinnitus *of any kind* (36%). Participants with prolonged spontaneous tinnitus were asked to report the affected ear(s).

#### Diagnosed Hearing Loss

Participants self-reported whether they had been diagnosed with hearing loss, defined as abnormal PTA thresholds. Since audiological jargon must be avoided, participants were asked, “*Have you ever been diagnosed with hearing loss? (By “hearing loss,” we mean abnormal results on a standard hearing test, which involves listening for quiet beeps.)*” Note that we did not constrain or record the context in which hearing loss was identified or the professional who identified it. If diagnosed hearing loss was reported, participants were asked when it developed and the affected ear(s).

#### Signs of Ear-Piece-Associated TTS

Ear-piece users were asked how often wearing an ear-piece led to signs of temporary hearing loss in the exposed ear (tinnitus and/or muffled hearing). Response options were “Never,” “Sometimes,” “About half the time,” “Most of the time,” and “Every time.”

### DIN Tasks

Participants were invited to perform online DIN testing via their web browser. They listened for digit triplets (“The digits… 3… 6… 8”) through their own headphones or earphones and entered them using an on-screen number pad. No details of the participants’ audio or computing equipment were recorded, nor measures of the quality of the listening environment. However, participants were instructed to listen in a quiet, distraction-free environment, and the “DIN Stimuli” and “DIN Quality Control” sections below describe the pretest calibration and troubleshooting procedures. Testing consisted of three blocks, using right-ear, left-ear, and antiphasic presentation of the digits (see “DIN Stimuli”). Testing was preceded by an unscored practice block using antiphasic presentation. The order of the right-ear and left-ear blocks was randomized. Of the 4,498 study participants, 3,752 chose to complete the listening tasks.

#### DIN Stimuli

Target sounds were random sequences of three digits sampled without replacement from the range 0 to 9, spoken by a female British-English talker. The digits were band-pass filtered between 0.12 and 8 kHz, to prevent listening performance being influenced by discrepancies in listeners’ headphones and earphones at extremely high or low frequencies. The carrier phrase and the digits were embedded in speech-spectrum-shaped Gaussian masking noise with the same long-term spectral shape as the digits.

In the test blocks with “right” and “left” presentation, both digits and masker were presented monaurally. In the “antiphasic” block, the masker was presented diotically (same waveform to both ears) while the digits were inverted in one ear (*S*_π_*N*_0_). In order to prevent loudness discomfort, the overall stimulus level (rather than the digit level or masker level) was held constant, and was determined by the participant in an initial subjective calibration stage. In this stage, they were presented alternately with a “loud” phrase and a “quiet” phrase, separated in root-mean-square level by 25 dB. They were instructed to adjust the volume control of their system until the “quiet” phrase was clearly audible and the “loud” phrase was loud but not uncomfortably so. All subsequent stimuli were presented at a level 5 dB below that of the “loud” phrase.

#### DIN Threshold Determination

Stimulus signal-to-noise ratio (SNR) varied adaptively, commencing each block at 2 dB and following a two-down, one-up stepping rule. Each trial with at least two out of three digits entered correctly was scored as correct. For the first two turnpoints, the SNR varied in 6-dB steps. For the final four turnpoints, the step size was 2 dB. Threshold was defined as the mean of the SNRs at the final four turnpoints. Participants were presented with visual feedback on their performance, to aid motivation.

#### DIN Quality Control

Care was taken to ensure that DIN data quality was not compromised by use of remote data collection techniques involving home computing and audio equipment. Prior to the task, the listener was instructed to check that test sounds were audible and that sounds intended for a specific ear were heard only in that ear. If these conditions were not met, troubleshooting tips were provided (e.g., checking and/or changing headphones/earphones and device audio settings); if these failed, the participant was asked to discontinue the test. As described in the “DIN Stimuli” section above, a subjective calibration procedure was designed to position DIN stimuli comfortably within the listener's dynamic range. Prior validation research with the DIN testing platform found that DIN data gathered at home using listeners’ own equipment corresponded closely to those gathered under laboratory conditions (Shehabi et al., 2025a) and that DIN thresholds predicted clinically measured PTA thresholds (Shehabi et al., 2025b). However, as acknowledged in the study protocol, exclusion of some DIN data on the basis of quality was also necessary, since some listeners might disregard instructions (e.g., persist with nonstereo equipment or listen through loudspeakers). Accordingly, data from 107 participants were excluded on the basis that one or more of their DIN thresholds were >3 standard deviations away from the sample mean. The remaining sample size for the speech-perception analyses was 3,645.

### Derivation of Variables

Given the large number of predictor and outcome variables in this study, comprehensive definitions are too extensive to be described in full here. These are given in the registered protocol (https://osf.io/4v59u/files) and are summarized below.

#### Ear-Piece Use (*DeviceUse*)

Whether the participant had ever regularly used an ear-piece (TRUE/FALSE).

#### Total Energy of Ear-Piece Noise Exposure (*DeviceNoise*)

This was calculated based on each participant's reported volume-control settings in quiet and noisy environments, and reported usage durations at each setting. Volume-control settings were converted to estimated sound levels using the mean ear-piece output-level measurements reported by ISVR Consulting (2010). (Note that the sound levels used to define the conversion function were those measured using a “normal” rather than a “raised” voice, and that the function accurately reflects the slightly nonlinear relation observed between volume and level.) Ear-piece usage durations were multiplied by 0.2 to yield estimated noise-exposure durations, since the proportion of time that speech/chatter is present in UK Police radio channels is relatively low (transmission-free periods and pauses in speech must be omitted). This proportion was found to be 29% for the Metropolitan Police Service (National Policing Improvement Agency, 2010) and 18% for West Yorkshire Police (West Yorkshire Police, 2021). ISVR Consulting (2010) suggested that a value of 15%–30% be used when calculating police noise exposure. We assumed a relatively conservative estimate of 20%. Sound levels and durations were combined into noise-exposure units linearly related to sound energy, which were summed across the career to yield *DeviceNoise*. One *DeviceNoise* unit equates to one working year of constant exposure (47 weeks × 40 h) at 85 dBA. Note that 85 dBA was selected as the basis for *DeviceNoise* units because it is the Upper Exposure Action Value specified in the Control of Noise at Work Regulations (Health and Safety Executive, 2005).

#### Weekly-Averaged Noise-Exposure Level (*DeviceWeeklyLevel*)

This variable was not defined in the registered protocol, but reflects the methods laid out in the Control of Noise at Work Regulations (Health and Safety Executive, 2005). Weekly-averaged noise-exposure level is one of the two metrics defined in the regulations for determining whether employee noise exposure is within prescribed limits and is used here for descriptive analysis only (RQ1). Inputs to the calculation are the total energy of ear-piece noise exposure over the career (*DeviceNoise*) and the reported number of working weeks of exposure over the career. The calculation assumes hypothetically that the individual was exposed to a constant sound level from the ear-piece throughout each working week, that is, for 40 h per week. Therefore, *DeviceWeeklyLevel* was calculated by averaging *DeviceNoise* over a duration equal to 40 × the number of weeks of exposure. The result is the sound level that, if present continuously for 40 h per working week in which an ear-piece was used, would lead to the same total noise-exposure energy as *DeviceNoise*.

#### Average Volume-Control Setting (*DeviceVolumeAverage*)

Effectively, this is a time-weighted average of the participant's volume-control settings, taking into account both the selected settings and the durations for which they were used over the career. Inputs to the calculation are the total energy of ear-piece noise exposure over the career (*DeviceNoise*) and the total duration of exposure over the career (the total number of hours in which sound was received through the ear-piece). The inverse of the formula used to obtain *DeviceNoise* (see above) was used to obtain the average sound level during the exposed period. Finally, this average sound level was converted to average volume-control setting, using the inverse of the conversion function described in the *DeviceNoise* section above.

#### Exposed Ear (*DeviceSide*)

For ear-piece users, this is whether the device was worn in the right or left ear. For nonusers, values were imputed: assigned pseudorandomly, such that the proportions of participants with “right” and “left” values were identical to those observed in ear-piece users (57% and 43%, respectively). Note that the imputed values are used to address RQ7 only, since this is the only analysis reliant on *DeviceSide* data from nonusers.

#### Proportion of Exposures Followed by Signs of TTS (*TTSProp*)

This is how often ear-piece users experienced signs of temporary hearing loss (tinnitus and/or muffled hearing) in the exposed ear after shifts spent wearing an ear-piece: “Never,” “Sometimes,” “About half the time,” “Most of the time,” or “Every time.”

#### Instances of Signs of Ear-Piece-Associated TTS (*TTSCount*)

This was estimated by combining *TTSProp* with the estimated number of shifts that the participant worked while wearing an ear-piece.

#### Ordinal TTS Measure (*TTSGroup*)

This was derived from *TTSCount* by dividing ear-piece users into three categories: those who had never experienced signs of ear-piece-associated TTS, those who reported experiencing them 1–100 times, and those who reported experiencing them >100 times.

#### Presence of Prolonged Spontaneous Tinnitus (*TinLong*)

This was defined as the presence or absence in the past 12 months of tinnitus that occurred spontaneously and lasted for over 5 min at a time. As already noted, this definition excludes other common forms of tinnitus, for example, tinnitus that lasted for under 5 min at a time and temporary tinnitus immediately following noise exposure.

#### Presence of Diagnosed Hearing Loss (*DiagnosedHL*)

This is the self-reported presence or absence of diagnosed hearing loss.

#### Exposed-Ear Deficit in DIN Performance (*DigitsDiff*)

This is the exposed-ear DIN threshold minus the control-ear DIN threshold. Note that lower DIN thresholds indicate better performance; therefore a speech-perception deficit in the exposed ear should lead to a positive value of *DigitsDiff*.

#### Antiphasic DIN Threshold (*DigitsAntiphasic*)

This is the threshold for dichotic DIN stimuli in which the phase of the digits was inverted between the two ears and the masking noise was diotic (*S*_π_*N*_0_).

#### Ear-Wise Tinnitus Variables (*TinModelEar*, *TinModelExposure*, and *TinModelPresence*)

For the RQ6 analysis only, data were analyzed ear-wise, with each observation representing an ear rather than a participant. In this reshaped version of the main study data, only unilateral ear-piece users with asymmetric tinnitus were included. The predictor variables were *TinModelEar* (whether the ear was the left or right) and *TinModelExposure* (whether the ear was the exposed ear or the control ear). The outcome variable was *TinModelPresence* (whether the ear was the predominant location of the participant's tinnitus).

#### Ear-Wise Diagnosed-Hearing-Loss Variables

These were defined as for the analogous tinnitus analysis above.

### Statistical Analysis

The analysis models corresponding to each RQ (defined in full in the registered protocol) are discussed below.

#### Ear-Piece Noise Exposure (RQ1)

The distribution of *DeviceWeeklyLevel*. The sample includes only ear-piece users. Values are compared with the “Upper Exposure Action Value” set out in the Control of Noise at Work regulations (2005).

#### Signs of TTS Following Exposure (RQ2)

The distribution of *TTSProp* responses among the five response options. The sample includes only ear-piece users.

#### Signs of TTS Versus Volume-Control Setting (RQ3)

Ordinal logistic regression model: *TTSProp* *∼* *DeviceVolumeAverage*.

The sample includes only ear-piece users. *Age* is to be included as a covariate if indicated.

#### Prolonged Spontaneous Tinnitus Versus Ear-Piece Use (RQ4)

Logistic regression model: *TinLong* *∼* *DeviceUse*.

The sample excludes participants who used an ear-piece nonunilaterally (i.e., did not consistently wear it in the same ear) and participants who chose to use one ear due to hearing loss or other pathology in the opposite ear. *Age* is to be included as a covariate if indicated.

#### Prolonged Spontaneous Tinnitus Versus Signs of TTS (RQ5)

Logistic regression model: *TinLong* *∼* *TTSGroup*.

The sample includes only ear-piece users. *Age* is to be included as a covariate if indicated.

#### Tinnitus Location Versus Ear-Piece Location (RQ6)

Logistic regression model: *TinModelPresence* ∼ *TinModelExposure* + *TinModelEar*.

The sample includes only ear-piece users with noncentral tinnitus (i.e., tinnitus that only or mainly affects one ear). It also excludes participants who used an ear-piece nonunilaterally (i.e., did not consistently wear it in the same ear) and participants who chose to use one ear due to hearing loss or other pathology in the opposite ear.

#### Exposed-Ear Speech-Perception Deficit Versus Ear-Piece Use (RQ7)

Linear regression model: *DigitsDiff* ∼ *DeviceUse* + *DeviceSide*.

The sample excludes participants who used an ear-piece nonunilaterally (i.e., did not consistently wear it in the same ear) and participants who chose to use one ear due to hearing loss or other pathology in the opposite ear. *EarTestedFirst is* to be included as a covariate if indicated.

## Results

### Ear-Piece Noise Exposure (RQ1)

**Figure 1.**
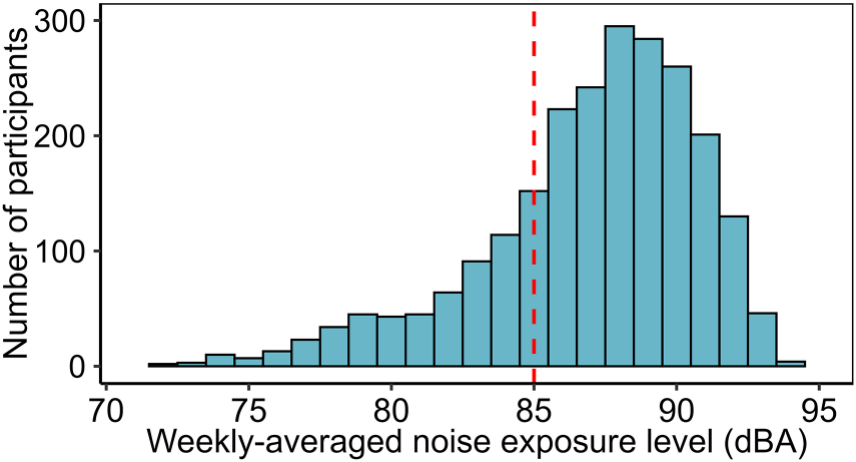
Estimated weekly-averaged ear-piece noise-exposure levels, calculated to allow exposures to be compared to the limits prescribed in the Control of Noise at Work Regulations (Health and Safety Executive, 2005). The red dotted line represents the Upper Exposure Action Value (85 dBA).

Figure 1 plots the distribution of values for weekly-averaged noise-exposure level (*DeviceWeeklyLevel*). As a reminder, calculation of this time-weighted average sound level assumes hypothetically that the individual was exposed to a constant sound level from the ear-piece throughout the working week, that is, for 40 h per week. *DeviceWeeklyLevel* had a median value of 87.7 dBA and exceeded 85 dBA (the Upper Exposure Action Value in the UK Control of Noise at Work regulations; Health and Safety Executive, 2005) for 75.8% of ear-piece users.

It is important to consider that *DeviceWeeklyLevel* is a metric designed solely to allow exposures to be compared with the limits set out in the Control of Noise at Work regulations, not a simple time-weighted average of exposure levels. In actuality, participants were exposed for far fewer than 40 h per week, because ear-pieces receive sound signals for only a small proportion of the time they are worn (National Policing Improvement Agency, 2010; West Yorkshire Police, 2021). Consequently, the median weekly exposure duration was 5.1 h and the median time-weighted-average sound level during periods of exposure was 96.8 dBA.

### Signs of TTS Following Exposure (RQ2)

Ear-piece users estimated how often they experienced signs of “temporary hearing loss” (defined as temporary tinnitus and/or muffled hearing in the exposed ear) after work shifts during which they wore an ear-piece. Table 2 provides the proportion of participants who selected each response option. Summarizing those self-report data, 45.2% of ear-piece users had experienced signs of temporary hearing loss in the exposed ear after a work shift, 5.5% after at least half of their work shifts, and 0.8% after every work shift.

**Table 2.** Reported Rates of Occurrence of TTS (Temporary Tinnitus and/or Muffled Hearing in the Exposed ear) After Ear-Piece Use.

Frequency of Occurrence of Signs of TTS in the Exposed Ear After Ear-Piece Use	Proportion of Ear-Piece Users (%)	95% CI
“Never”	54.8	52.7 to 56.9%
“Sometimes”	39.8	37.7 to 42.0%
“About half the time”	1.7	0.0 to 3.8%
“Most of the time”	3.0	0.9 to 5.1%
“Every time”	0.8	0.0 to 3.0%

*Note*. TTS = temporary threshold shift. 95% confidence intervals (CIs) were computed using the Sison–Glaz method, which provides simultaneous CIs for all categories while controlling the overall error rate.

### Signs of TTS Versus Volume-Control Setting (RQ3)

**Figure 2.**
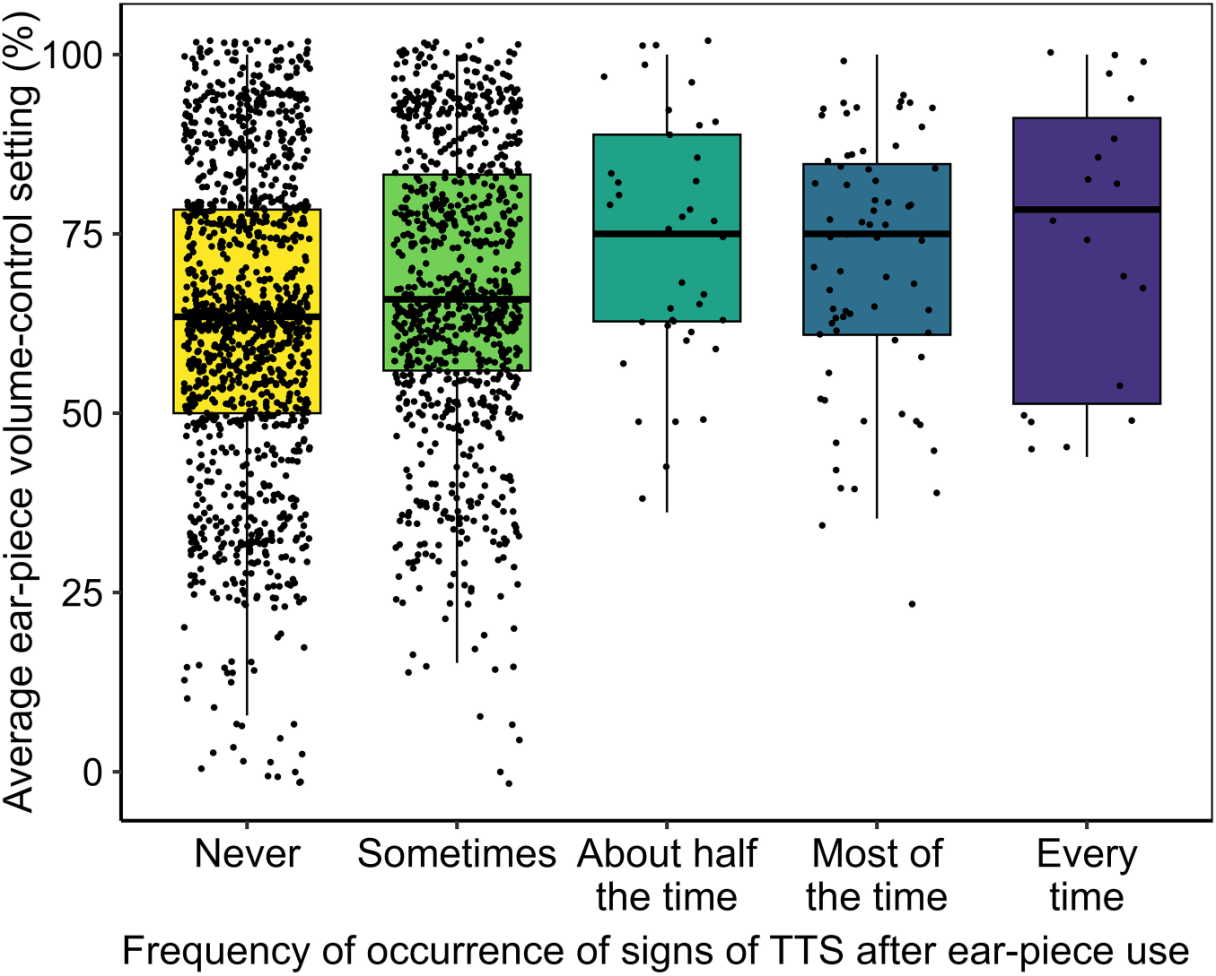
Weighted-average volume-control settings for five groups of ear-piece users, divided based on their reported frequency of occurrence of signs of ear-piece-associated TTS. Within each boxplot, the thick horizontal line represents the median, the box the interquartile range (IQR), the lower whisker the 25th percentile minus 1.5 × IQR, and the upper whisker the 75th percentile plus 1.5 × IQR. TTS = temporary threshold shift.

Figure 2 plots the relation between average ear-piece volume-control setting (*DeviceVolumeAverage*) and frequency of occurrence of ear-piece-associated TTS (*TTSProp*). Analysis via ordinal logistic regression confirmed that higher volume-control settings were associated with more frequent signs of TTS (*t* = 6.25, *P* < .00001). Exploratory versions of the analysis model controlling for age and sex are reported in the Supplemental materials; null effects were observed for these demographic factors and the effect of volume-control setting remained robust (*P* < .00001).

### Prolonged Spontaneous Tinnitus Versus Ear-Piece Use (RQ4)

**Figure 3.**
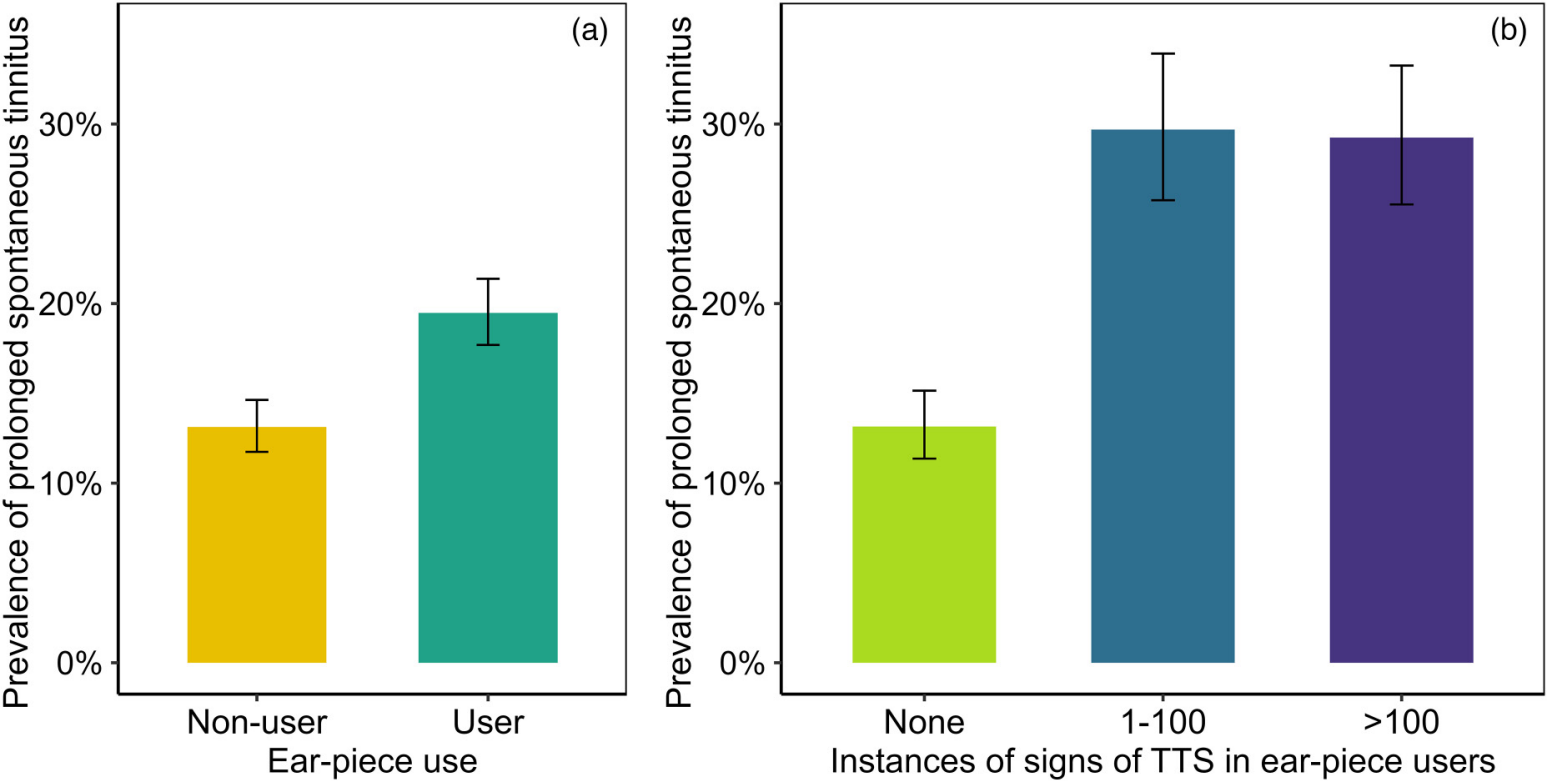
Prevalence of prolonged spontaneous tinnitus. (a) In ear-piece users vs. nonusers. (b) In three groups of ear-piece users: those who had not experienced signs of ear-piece-associated TTS, those who reported experiencing them on 1–100 occasions, and those who reported experiencing them on >100 occasions. Note that error bars represent unadjusted 95% confidence intervals, calculated using a binomial approximation based on the observed proportions, and not adjusted intervals obtained from regression analysis. TTS = temporary threshold shift.

Figure 3a shows the prevalence of prolonged spontaneous tinnitus for ear-piece users (19.5%) and nonusers (13.1%). Following the registered protocol, potential relations with age were also explored, to inform the analysis model. Ear-piece users were 2.0 years younger than nonusers (95% confidence interval [CI] 1.5 to 2.5, *P* < .00001) and participants with tinnitus were 1.9 years older than those without (95% CI 1.2 to 2.5, *P* < .00001); hence, age was included in the multiple logistic regression model. Results confirmed that ear-piece use (*DeviceUse*) was associated with an increased risk of tinnitus (*TinLong*), controlling for age (odds ratio [OR] 1.7, 95% CI 1.5 to 2.1, *P* < .00001). As reported in the Supplemental materials, the effect of ear-piece use remained robust when sex was added as a covariate (*P* < .00001).

### Prolonged Spontaneous Tinnitus Versus Signs of TTS (RQ5)

Figure 3b illustrates the prevalence of prolonged spontaneous tinnitus for three groups of ear-piece users: those who had never experienced signs of ear-piece-associated TTS (13.2% prevalence), those who reported experiencing them 1–100 times (29.7% prevalence), and those who reported experiencing them >100 times (29.2% prevalence). Comparing the leftmost bars in Figure 3a and b, tinnitus prevalence for ear-piece users without TTS was almost identical to that observed for nonusers of ear-pieces (13.1%).

The apparent association between the instances of TTS and risk of tinnitus was analyzed formally via multiple logistic regression, with *TTSGroup* as the explanatory variable and *TinLong* as the outcome. Following the registered protocol, potential relations with age were explored, to inform the analysis model. Participants with 1–100 instances of signs of TTS did not differ in age from those without (*P* = .19) but those with >100 instances were 2.3 years younger than those without (95% CI 1.5 to 3.1, *P* < .00001); hence, age was added as a covariate.

Results indicated that tinnitus risk was substantially increased by experiencing signs of ear-piece-associated TTS on 1–100 occasions (OR 2.8, 95% CI 2.2 to 3.6, *P* < .00001) or on >100 occasions (OR 3.0, 95% CI 2.4 to 3.9, *P* < .00001), controlling for age. As is clear from Figure 3b and the overlapping CIs, moving from the “1–100” category to the “>100” category was not associated with a further increase in tinnitus risk. As reported in the Supplemental materials, the pattern of results was not altered by adding sex as a covariate.

### Tinnitus Location Versus Ear-Piece Location (RQ6)

**Figure 4.**
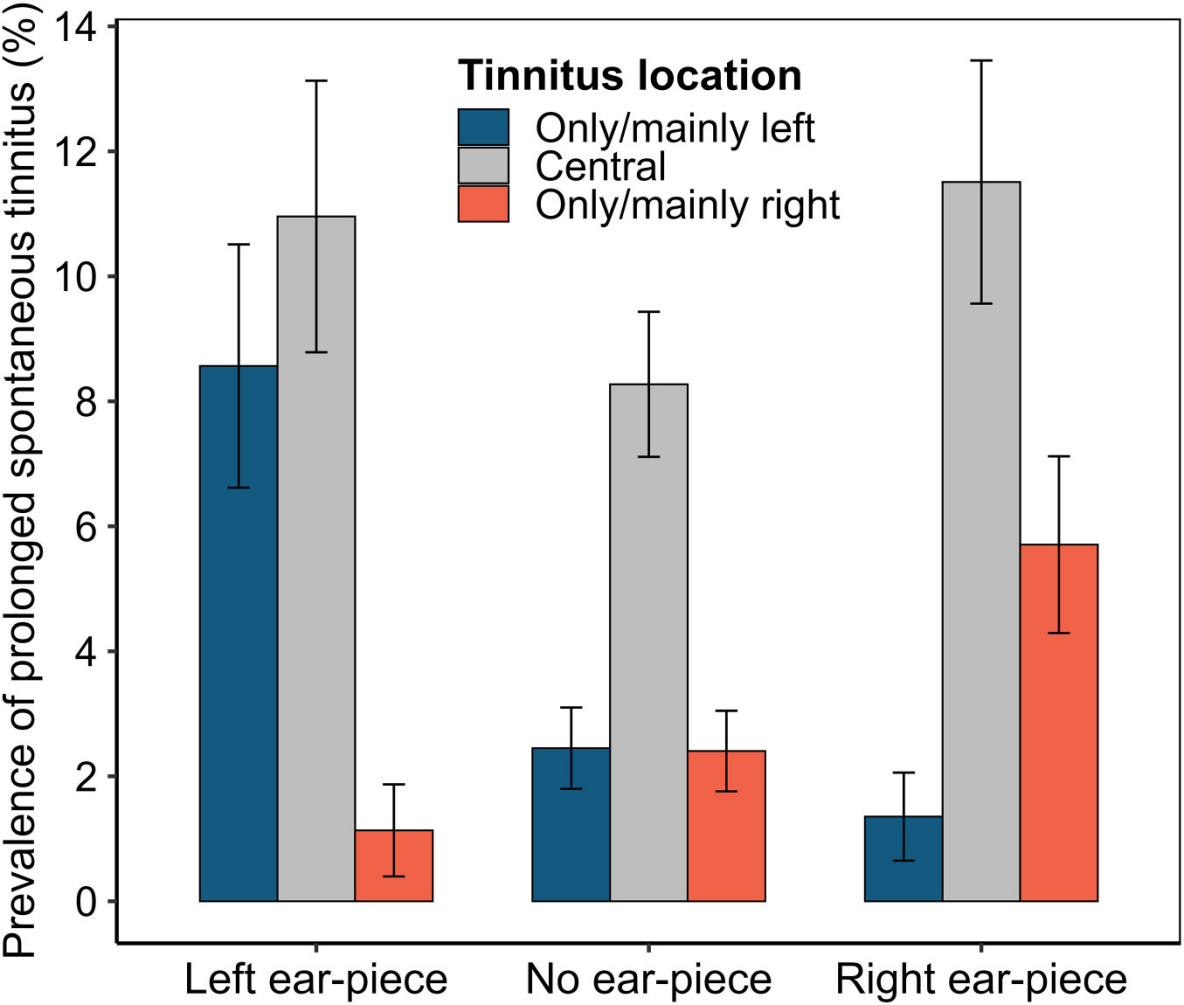
Prevalence of three locations of prolonged spontaneous tinnitus (central, only/mainly right, and only/mainly left) for three exposure groups (non-ear-piece users, right-sided ear-piece users, and left-sided ear-piece users). Note that error bars represent unadjusted 95% confidence intervals, calculated using a binomial approximation based on the observed proportions, and not adjusted associations obtained from regression analysis.

Figure 4 shows the prevalence of three locations of prolonged spontaneous tinnitus (central, only/mainly right, and only/mainly left) for three exposure groups (non-ear-piece users, right-sided ear-piece users, and left-sided ear-piece users). The tendency for asymmetric tinnitus on the side of exposure is clearly apparent, though central tinnitus was the most common form of tinnitus for all three groups. Analysis included only ear-piece users with asymmetric tinnitus (the leftmost and rightmost blue and red bars in Figure 4). A mixed-effects logistic regression model was used to test for an effect of ear-piece location (*TinModelExposure*) on tinnitus location (*TinModelPresence*), controlling for ear (*TinModelEar*). Note that ear was controlled in case the left or right ears were more prone to tinnitus, regardless of ear-piece exposure (Axelsson & Ringdahl, 1989). The results indicate a nonsignificant trend toward left-sided tinnitus (*P* = .07) and a highly significant association between ear-piece location and tinnitus location (*P* < .00001).

### Exposed-Ear Speech-Perception Deficit Versus Ear-Piece Use (RQ7)

**Figure 5.**
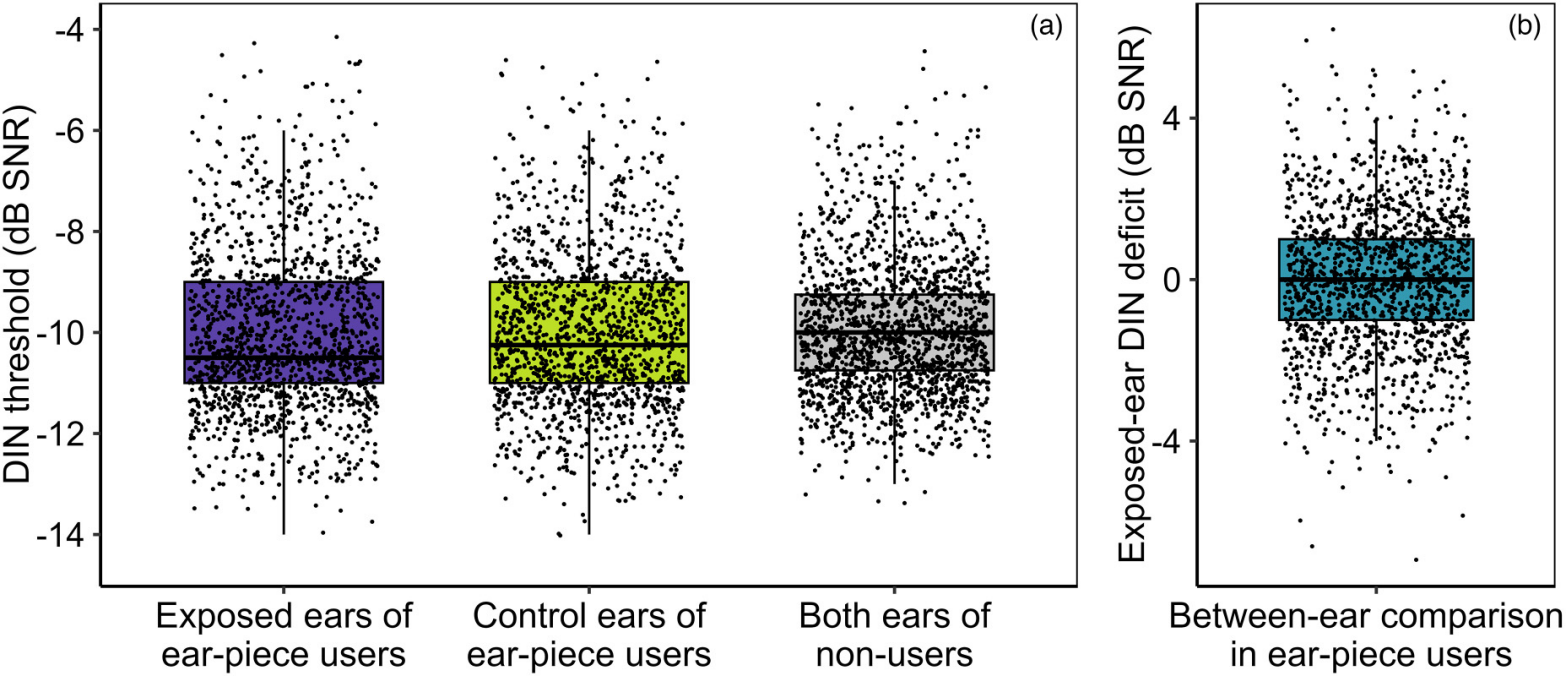
Boxplots of DIN measures. Within each, the thick horizontal line represents the median, the box the interquartile range (IQR), the lower whisker the 25th percentile minus 1.5 × IQR, and the upper whisker the 75th percentile plus 1.5 × IQR. (a) Monaural DIN thresholds for the exposed and control ears of ear-piece users, along with the both-ear-average DIN thresholds of nonusers. (b) Values of the between-ear difference measure (DigitsDiff) in ear-piece users. Note that positive values would be expected if exposed ears performed more poorly than control ears. DIN = Digits-In-Noise.

Figure 5a plots DIN thresholds from the ear-piece-exposed and control ears of ear-piece users and both ears of nonusers. The coinciding distributions suggest that ear-piece use is unlikely to be associated with deficits in speech perception in the exposed ear relative to the control ear.

Formal statistical analysis required a more sophisticated approach, since DIN thresholds might be lower in the right ear, regardless of exposure (Hugdahl, 2011). To address possible confounding, the model compared thresholds between the exposed ear and the control ear in ear-piece users and nonusers, while also controlling for a potential main effect of ear (right/left). To allow this approach, the “side of exposure” was imputed for nonusers of ear-pieces (see the “Derivation of Variables” section).

A linear regression model was fitted, with outcome variable *DigitsDiff* (the exposed-ear deficit in DIN threshold, i.e., the exposed-ear DIN threshold minus the control-ear DIN threshold; Figure 5b). Explanatory variables were *DeviceUse* (user/nonuser) and *DeviceSide* (right/left). Following the registered protocol, preliminary analysis also tested for potential order effects (since the ear tested last might be aided by practice); none were evident, so testing order was omitted from the model.

Contrary to our hypothesis, *DigitsDiff* did not differ between ear-piece users and nonusers (*P* = .49), controlling for *DeviceSide*. The mean value of *DigitsDiff* in ear-piece users was 0.0 dB SNR (95% CI −0.1 to 0.1 dB SNR); as noted previously, a positive value would be expected if the exposed ear performed more poorly than the control ear.

Results of exploratory DIN analyses are reported in the “Exploratory Analyses: DIN Thresholds” section of the Supplemental materials. These include models with antiphasic DIN thresholds as the outcome variable and models with TTS or total energy of noise exposure as the explanatory variable. Most produced null results, with one exception. Participants who experienced the signs of ear-piece associated TTS on >100 occasions had slightly higher antiphasic hearing thresholds than participants reporting no TTS, controlling for age (*B* = 0.57 dB, 95% CI 0.29 to 0.86 dB, *P* = .00008).

## Discussion

### Estimated Ear-Piece Exposures Exceed Recommended Levels

Estimated weekly-averaged noise-exposure levels from ear-pieces were high: the median value was 88 dBA and the 90th percentile was 91 dBA. The Upper Exposure Action Value in the UK Control of Noise at Work regulations (Health and Safety Executive, 2005) is 85 dBA, above which employers are generally required to take “reasonably practicable” steps to reduce exposure. As discussed in “Limitations” section below, weekly-averaged noise-exposure level is a composite estimate, incorporating values estimated by participants and average values derived from past measurements of TETRA devices and transmissions. It is therefore possible that levels and/or durations have been overestimated. However, unless very substantial overestimation has taken place, the distribution of overall exposures remains concerning. Reduction of estimated weekly-averaged noise-exposure level to 85 dBA or less for 90% of participants would require a 6-dB decrease in sound levels or a fourfold decrease in exposure durations.

Consistent with these results is the more straightforward finding that participants reported using high volume-control settings. The mean reported setting in noisy environments was 81% of full volume, and users reported selecting such settings 48% of the time; the mean setting in quieter environments was 51%. These values are at odds with the low volume-control settings recommended for hearing health preservation (Health and Safety Laboratory, 2004; ISVR Consulting, 2010). The reasons for police personnel selecting such high settings should be investigated, allowing for both acoustic and behavioral factors. The recent Police National Hearing Survey (Shoetan et al., 2024) provided some preliminary qualitative data on the issue: radio transmissions were described by some as being “too soft” or “muffled” and many users reported that they consequently “needed their earpieces at full volume.” See “Implications and Next Steps” section below for further discussion.

### Ear-Piece Use can Lead to Signs of TTS, Especially at High Volume-Control Settings

Almost half (45.2%) of ear-piece users reported experiencing signs of ear-piece-associated TTS. More striking still is the finding that a sizeable minority (5.5%) experienced this phenomenon after half or more of their ear-piece exposures. Auditory science increasingly recognizes that TTS may be less benign than once thought, even if its overt perceptual effects resolve entirely. It may be associated with permanent subclinical damage to cochlear hair cells and/or auditory nerve fibers, which may accumulate and eventually present as bothersome hearing symptoms (Plack et al., 2014).

The results for RQ3 suggest that the subset of ear-piece users with frequent signs of ear-piece-associated TTS selected high volume-control settings, even by comparison with the cohort as a whole. High settings appear to be necessary but not sufficient for frequent signs of TTS, a pattern consistent with individual differences in susceptibility to TTS. However, given the observational study design, it is important to avoid assumptions about causative direction. It remains possible that TTS leads to a need for high volume-control settings, rather than high volume-control settings leading to TTS. Moreover, a number of psychosocial factors may influence TTS reporting, such as noise sensitivity and health anxiety. Nonetheless, both the prevalence of frequent signs of TTS and the high volume-control settings reported by the cohort are concerning.

### Ear-Piece Use is Associated With Persistent Tinnitus

Ear-piece use was associated with a 73% increase in risk of prolonged spontaneous tinnitus, a potentially distressing condition that can significantly affect quality of life, mental health, and daily functioning (National Institute for Health and Care Excellence, 2020). It is important not to draw firm conclusions based on this finding alone, due to risk of confounding. Ear-piece users may differ from nonusers in terms of demographic, health, environmental, and behavioral factors that also influence tinnitus risk. Risk of confounding is particularly acute because ear-piece users predominantly occupy operational roles, whereas nonusers tend to be in nonoperational positions. A posthoc analysis of job-role data indicated that only 19.7% of police “staff” (nonoperational) used an ear-piece, compared with 62% of currently and formerly operational personnel (e.g., constables, sergeants, inspectors, chief inspectors, and superintendents). Although controlling for age and sex did not weaken the association, other hidden confounders likely remain.

The RQ6 analysis is therefore paramount, since it capitalizes on between-ear comparisons, testing for an association between tinnitus location and ear-piece location. Effectively, each participant served as their own control, thereby controlling practically all exogenous and endogenous confounding variables. The results indicated that tinnitus location was strongly associated with the side of exposure: asymmetric tinnitus tended to be localized in the ear with the ear-piece. Note that care was taken to ensure that the results were not influenced by reverse causation; participants were excluded from the analysis if they reported wearing their ear-piece in one ear due to hearing pathology in the other.

In combination, the RQ4 and RQ6 findings present powerful evidence of association between ear-piece use and tinnitus. Note that a very similar pattern of results was observed for self-reported diagnosed hearing loss (Supplemental materials), that is, higher risk of hearing loss in ear-piece users than nonusers and localization of symptoms to the exposed ear.

### The Association Between Ear-Piece Use and Tinnitus Risk is Confined to Users Who Experience Signs of TTS

It appears that the association between ear-piece use and tinnitus risk was driven by the subset of ear-piece users who experienced signs of TTS after use. The prevalence of tinnitus for ear-piece users without TTS (13.2%) was almost identical to that observed for nonusers (13.1%), but more than doubled for users who did experience signs of TTS (29.5%). Given the strength of this finding, readers might reasonably question whether artifactual explanations—such as flaws in the survey wording—could account for the association. If participants were unsure as to the distinction between *prolonged spontaneous tinnitus* and *TTS-related tinnitus*, a trivial association between these variables could result. We provide the full questionnaire wording in the Supplemental materials, to make clear the methods used to carefully distinguish these two phenomena.

Consistent with the above finding, Brungart et al. (2025) report associations between TTS frequency and self-reported persistent hearing difficulties for a sample of 10,492 members of the US military, even when PTA was normal. Collectively, these results have important implications for hearing health conservation, not only in the UK Police but in noise-exposed populations more generally. A noise exposure that induces TTS in an individual should perhaps be regarded as a source of risk for that individual, even if peers or coworkers are unaffected. Given individual differences in susceptibility to noise damage, TTS may be a valuable early warning sign of accumulating auditory damage in the individual.

### DIN Performance is Insensitive to All Exposure Metrics

If DIN measures were sensitive to ear-piece-induced hearing changes, they could form simple and low-cost components of occupational hearing health surveillance. Evaluable DIN data were gathered from 3,645 participants in the present study, at almost zero marginal cost, using at-home listening equipment and a browser-based testing platform. Naturally, DIN thresholds measured at home rely on consumer audio and computing equipment of varying quality and may additionally be influenced by background noise and distractions in the environment, none of which were recorded in the present study. However, prior evaluation research conducted with this platform found good agreement between at-home and laboratory DIN data, both in terms of threshold SNRs and absolute test–retest differences (Shehabi et al., 2025a).

The present research revealed no relations between DIN measures and noise exposure. This was true for both monaural and antiphasic versions of the DIN test, and regardless of whether exposure was represented by a simple binary measure (user/nonuser) or by total energy of noise exposure. Instances of self-reported TTS were not associated with the between-ear DIN difference measure, though they showed a small but highly significant association with antiphasic DIN thresholds (*B* = 0.57 dB, *P* = .00008; see Supplemental materials).

The diverse potential explanations for these generally null results should be considered. One interpretation is that DIN thresholds are relatively insensitive to noise-induced hearing pathology resulting from ear-piece exposure. Speech-in-noise perception draws on cognitive and central processes (Mattys et al., 2012), which can obscure subtle peripheral deficits. For the specific purpose of detecting cochlear synaptopathy, DiNino et al. (2022) argue that tasks should use challenging SNRs, minimal semantic context, and strong reliance on temporal cues. Our antiphasic DIN variant partially meets these criteria but was not explicitly designed for synaptopathy detection. In relation to extended-high-frequency hearing loss, evidence for effects on speech-in-noise performance is mixed (Lough & Plack, 2022). In contrast, losses within the conventional PTA range (<8 kHz) could affect DIN performance (Shehabi et al., 2025b) and are plausible in our sample. The median age was 56 years, participants had up to 24 years of ear-piece exposure, and a substantial minority reported diagnosed hearing loss, which was found to relate to ear-piece use and TTS (see Supplemental materials). This discrepancy points to a potentially intractable problem for the use of speech-in-noise tasks to assess ear-piece-related dysfunction. Crucially, an ear that receives spoken messages through an ear-piece for years or decades may undergo an “auditory training” effect, enhancing its processing of speech in background noise (Moore & Amitay, 2007). The benefits of this “auditory training” for DIN performance may counteract the detrimental effects of noise exposure, negating the use of speech-in-noise testing in this context.

It is important to also consider more skeptical interpretations. All of the positive study findings rest on self-report measures, and are subject to memory limitations and bias. Null results of the DIN analysis may therefore throw into question the remaining study results. However, the latter are characterized by very strong effects that cannot readily be dismissed; the authors consider that in-person testing to assess ear-piece-induced auditory asymmetry should be a priority.

### Limitations

The study focused on ear-piece noise exposures, which supported between-ear comparisons. However, effects of these exposures may be harder to detect among participants with additional bilateral noise exposure from recreational and/or occupational sources. Policing often involves exposure to a great number of environmental noise sources, such as traffic, crowds, and sirens.

Noise-exposure levels and durations were not measured directly. Our estimates of each are dependent on both self-report data (volume-control settings and durations of ear-piece wear) and average values derived from past measurements (ear-piece output levels and durations of transmitted noise). Ear-pieces differ across police forces and even between roles within a single force, and lack of data on the specific models used by individuals prevented control of these differences in our analysis. Ultimately, the analytical approach was relatively crude: using mean output levels from a large sample of ear-pieces to convert volume-control settings to estimated sound levels. Similarly, duration of transmitted noise is likely to vary across forces and between individuals. The National Policing Improvement Agency (2010) estimated that noise/chatter is present on a typical radio channel 29% of the time, based on Metropolitan Police recordings; West Yorkshire Police puts the value at 18%; our analysis assumed 20%. However, conservatively reducing our exposure values to align with the West Yorkshire estimate would still leave most respondents with a weekly-averaged noise-exposure level exceeding 85 dBA.

Self-reported hearing data are also limited by human fallibility. Self-selection bias is a key concern: individuals with auditory symptoms that could feasibly be related to exposure might be more motivated to take part in the study. Bias could be introduced by shared method variance and subjective interpretation; responses could be biased by the belief that auditory symptoms are related to exposure, especially in the ear that wore the ear-piece. Noise sensitivity could act as a confounding factor, influencing the reporting of exposure habits, tinnitus, and signs of TTS. Moreover, as emphasized previously, the data are strictly observational, preventing firm conclusions regarding causative direction.

The analyses reported in this study are restricted to those defined in the preregistered protocol, in order to avoid conflating exploratory and confirmatory inference and to maintain a focused primary report. That said, other exploratory approaches are possible, and the de-identified dataset will be made openly available at https://osf.io/4v59u/files upon publication of the present article.

### Implications and Next Steps

Ear-piece exposure appears to be associated with tinnitus and hearing loss, but the results are based solely on self-report, with all its inherent limitations. A valuable next step would be recruitment of participants for in-person testing. Between-ear comparisons should again be conducted, this time incorporating a wide array of physiological and behavioral measures, to test for hearing asymmetry and identify site(s) of damage along the auditory pathway.

Some findings of the present study are consequential even in the absence of laboratory testing, and suggest that it may be worthwhile to consider strategies for reducing risk to police hearing health. An important question is why officers, by their own testimony, are not selecting the low volume-control settings that should guard against accumulating hearing damage. Adequate answers are likely to require both detailed examination of device performance and in-depth qualitative data from users. Collectively, this information could inform risk-reduction measures.

Ultimately, risk reduction might involve retraining/awareness, but potentially also device design. The Police National Hearing Survey found that 54.8% of radio users reported issues hearing police radio equipment (Shoetan et al., 2024). In some police working environments, limited intelligibility may be intractable, for example, crowd control at a 100-dBA football match. Moreover, since operational personnel need to maintain good awareness of environmental sounds, most ear-pieces (excepting those designed for firearms and motorcycle officers) are nonoccluding (ISVR Consulting, 2010). It is important to recognize that choosing device settings that balance comprehension with hearing health is inherently challenging, especially given the high stakes involved in policing. Some messages are safety-critical, and the safety risks of not hearing them in noisy environments must be weighed against the long-term risks of hearing loss (ISVR Consulting, 2010). Nonetheless, given the rapid pace of technological improvement, possibilities may exist for reducing device loudness while maintaining or improving intelligibility, such as acoustic filtering and/or active noise cancellation to optimize the balance of transmitted versus environmental sound.

Optimal device performance is also unlikely to be one-size-fits-all, especially given individual differences in susceptibility to hearing damage. Identifying individual staff at risk is therefore important. Our data and those of Brungart et al. (2025) suggest that TTS may serve as an important warning sign on an individual basis. Additionally, expansion of more conventional methods of hearing health surveillance may be warranted. Regular PTA is currently routine for only a minority of police employees, for example, firearm officers, while most of the remainder are tested only as new recruits. Of course, the unique dangers of firearm noise exposure are well established, due to an extensive body of research (Sonstrom Malowski et al., 2022). Until recently, the risks of ear-piece exposure have been given no such attention by the auditory research community. As they are brought into focus, it may be necessary to consider whether hearing surveillance is appropriate for wider groups of police personnel.

## Conclusions

Police personnel who used ear-pieces reported selecting high volume-control settings, despite long-standing recommendations to use these devices at low volumes to protect against hearing damage. Our estimates of resulting exposure levels are relatively crude, but may warrant attention because, for many participants, levels substantially exceeded the 85 dBA Upper Exposure Action Value. Nearly half of ear-piece users reported experiencing signs of TTS in the exposed ear after use, with 5.5% reporting this phenomenon after half or more of their ear-piece exposures.

Ear-piece use was associated with 73% increased risk of tinnitus. Tinnitus location tended toward the exposed ear, suggesting that the association is specific to ear-piece use rather than attributable to confounding factors. Notably, the association between ear-piece use and tinnitus was driven by the subset of users who experienced signs of TTS. Exploratory analyses (reported in the Supplemental materials) revealed that self-reported diagnosed hearing loss exhibited similar patterns of association with ear-piece use and TTS as did tinnitus. No associations were observed between ear-piece use and performance on online listening tasks; the sole positive DIN finding was a small exploratory association between the signs of TTS and antiphasic listening performance.

The results suggest that it may be important to conduct in-person auditory testing to determine and characterize ear-piece-related auditory pathology. In terms of police hearing health, additional work may be needed to explore and address the potential effects of ear-piece noise exposure; these may include updated approaches to risk assessment, risk reduction, and hearing health surveillance. More broadly, our findings on the role of TTS in the development of persistent hearing symptoms have implications for monitoring hearing health in wider noise-exposed populations.

## Supplemental Material

sj-docx-1-tia-10.1177_23312165251410988 - Supplemental material for Leveraging Monaural Exposures to Reveal Early Effects of Noise: Evidence from Police Radio Ear-Piece Use Supplemental material, sj-docx-1-tia-10.1177_23312165251410988 for Leveraging Monaural Exposures to Reveal Early Effects of Noise: Evidence from Police Radio Ear-Piece Use by Hannah Guest, Paul Elliott, Martie van Tongeren, Joseph Laycock, Steven Thorley-Lawson, Michael A. Stone, Michael T. Loughran and Christopher J. Plack in Trends in Hearing

## Notes

1. Note that the study research questions and analysis models are presented in a different order in the present study than in the protocol, but their substance is unaltered.
