# Supplementary material for: Leveraging Monaural Exposures to Reveal Early Effects of Noise: Evidence from Police Radio Ear-Piece Use
Source: Trends Hear. 2026 Jan 30;30:23312165251410988. doi: 10.1177/23312165251410988 (PMC12858745; doi:10.1177/23312165251410988)
Supplement: sj-docx-1-tia-10.1177_23312165251410988 - Supplemental material for Leveraging Monaural Exposures to Reveal Early Effects of Noise: Evidence from Police Radio Ear-Piece Use [file sj-docx-1-tia-10.1177_23312165251410988.docx]

Exploratory analyses

## DIN thresholds

The primary DIN analysis, reported in the main paper, tested for deficits in exposed-ear DIN thresholds associated with ear-piece use. We added exploratory DIN analyses employing an alternative DIN measure (antiphasic DIN threshold, *DigitsAnti*) and/or an alternative noise-exposure measure (total energy of ear-piece noise exposure, *DeviceNoise*). A multiple linear regression model was constructed to test for an association between *DeviceUse* and *DigitsAnti*, controlling for age (see Figure SM1). Two further models testing for: an association between log(*DeviceNoise*) and *DigitsDiff*, controlling for age; and an association between log(*DeviceNoise*) and *DigitsAnti*, controlling for age. No significant associations were observed (*p* > 0.05).


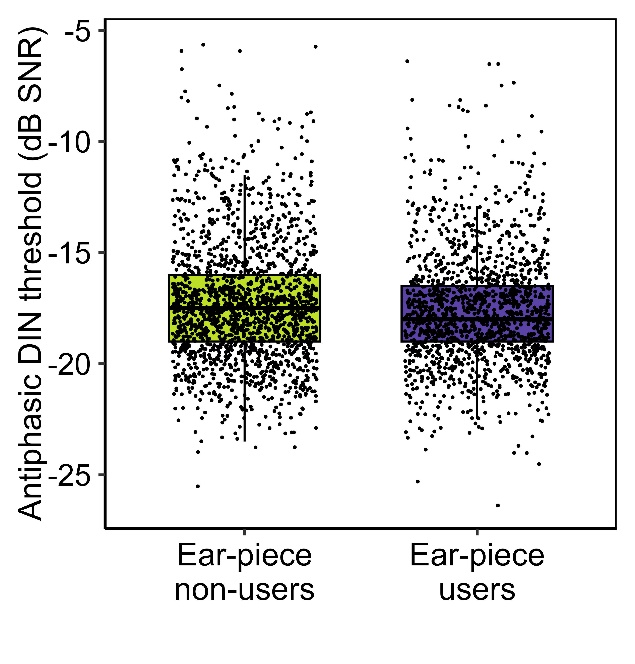


##### Figure SM1. Antiphasic DIN thresholds for ear-piece users and non-users. Within each boxplot, the thick horizontal line represents the median, the box the interquartile range (IQR), the lower whisker the 25th percentile minus 1.5 * IQR, and the upper whisker the 75th percentile plus 1.5 * IQR.

We also tested for associations between signs of TTS and DIN outcome measures, controlling for age, analogous to the RQ5 analysis in the main paper. Figure SM2 plots values of the two DIN measures (*DigitsDiff* and *DigitsAnti*) in relation to age and separated into three groups: participants who had never experienced signs of ear-piece-associated TTS, those who reported experiencing them 1-100 times, and those who reported experiencing them >100 times. Two multiple linear regression models were constructed, testing for: an association between *TTSGroup* and *DigitsDiff*, controlling for age; and an association between *TTSGroup* and *DigitsAnti*, controlling for age. Results of the *DigitsDiff* model were resoundingly null (all *p* > 0.05), but not so for *DigitsAnti*. Antiphasic DIN thresholds were significantly higher for participants reporting signs of ear-piece-associated TTS on >100 occasions compared with those reporting none, controlling for age (*B* = 0.57 dB, 95% CI = 0.29 to 0.86 dB, *p* = 0.00008). The contrast for participants reporting 1-100 occasions did not reach significance (*B* = 0.25 dB, 95% CI = -0.05 to 0.56 dB, *p* = 0.10).


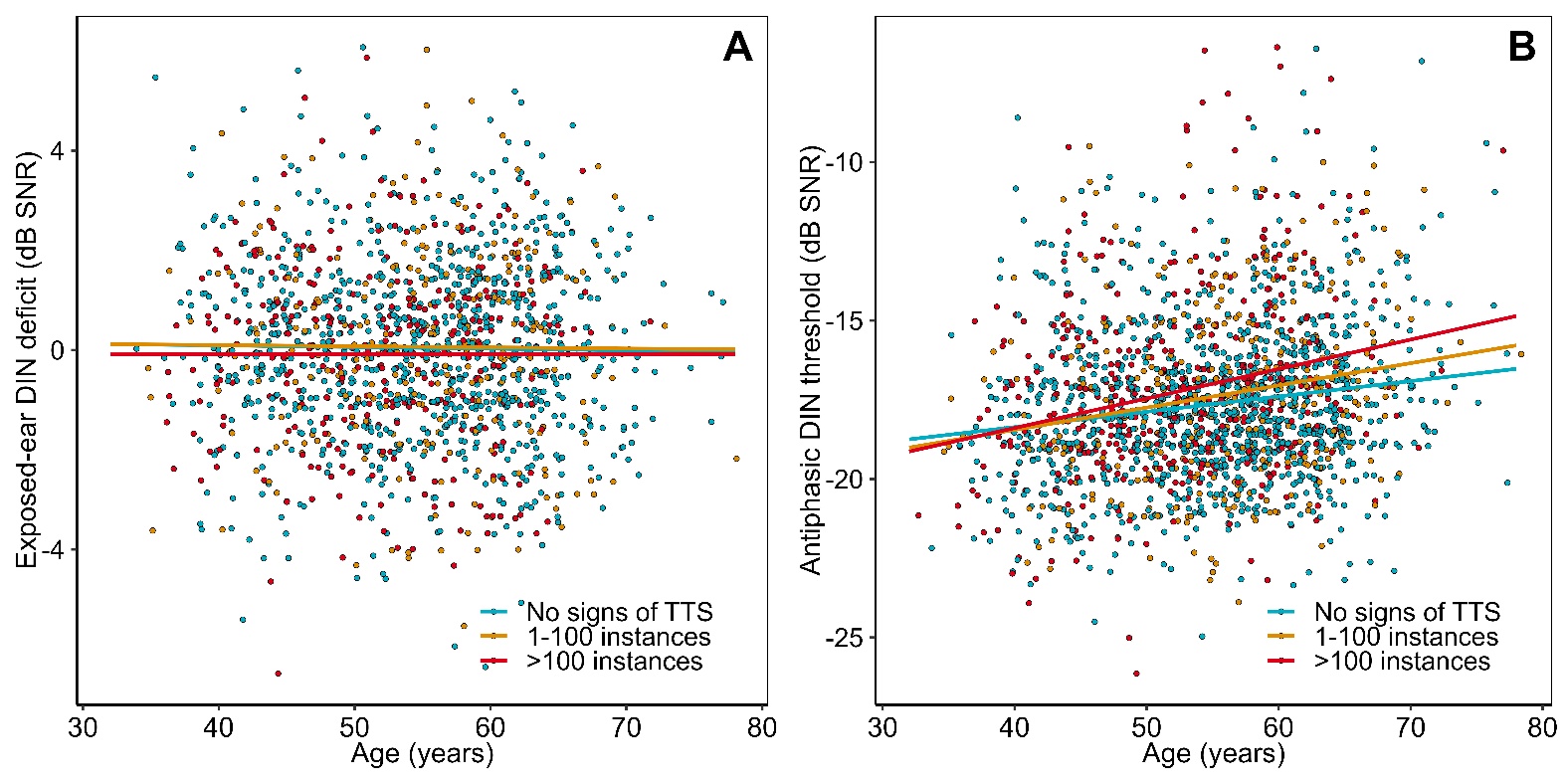


##### Figure SM2. DIN outcome measures vs age, separated into three groups of ear-piece users: those who had not experienced signs of ear-piece-associated TTS, those who reported experiencing them on 1-100 occasions, and those who reported experiencing them on >100 occasions. A: The exposed-ear DIN deficit (DigitsDiff). B: Antiphasic DIN thresholds (DigitsAnti).

## Diagnosed hearing loss

Supplementary analyses were conducted to test for associations with diagnosed hearing loss. Note that all hearing-loss data were obtained by self-report only (participants were asked if they had been *“diagnosed with hearing loss”*, with *“hearing loss”* being defined explicitly as *“abnormal results on a standard hearing test, which involves listening for quiet beeps”*). Figure SM3A displays the prevalence of self-reported diagnosed hearing loss (*DiagnosedHL*) for ear-piece users (12.1%) and non-users (8.3%). Multiple logistic regression confirmed higher prevalence for ear-piece users, controlling for age (OR = 1.8, 95% CI = 1.4 to 2.2, *p* < 0.00001).

We also tested for an association between signs of TTS and risk of self-reported diagnosed hearing loss, controlling for age, analogous to the RQ5 analysis in the main paper. Figure SM3B illustrates hearing-loss prevalence for three groups of ear-piece users: those who had never experienced signs of ear-piece-associated TTS (8.8% prevalence), those who reported experiencing them 1-100 times (14.7% prevalence), and those who reported experiencing them >100 times (17.1% prevalence). Prevalence was substantially higher for those who reported experiencing signs of ear-piece-associated TTS on 1-100 occasions (OR = 1.8, 95% CI = 1.3 to 2.4, *p* = 0.0004) or on >100 occasions (OR = 2.6, 95% CI = 1.9 to 3.5, *p* < 0.00001), controlling for age. The increase in risk associated with moving from the “1-100” category to the “>100” category was marginally significant (*p* = 0.03, uncorrected for multiple comparisons).

Analogous to the tinnitus data presented in the main paper, Figure SM3C plots the prevalence of self-reported diagnosed hearing loss categorised based on laterality (affecting both ears equally, only/mainly the right ear, or only/mainly the left ear) for three exposure groups (non-ear-piece users, right-sided ear-piece users, and left-sided ear-piece users). The tendency for poorer self-reported hearing on the side of exposure is clearly apparent. The model specification is identical to that employed for the RQ6 analysis, substituting diagnosed hearing loss for tinnitus. The results indicate a highly significant association between ear-piece location and the self-reported poorer-hearing ear (*p* < 0.00001). There is also a trend for poorer self-reported hearing on the left, controlling for ear-piece location (*p* = 0.006, uncorrected for multiple comparisons).


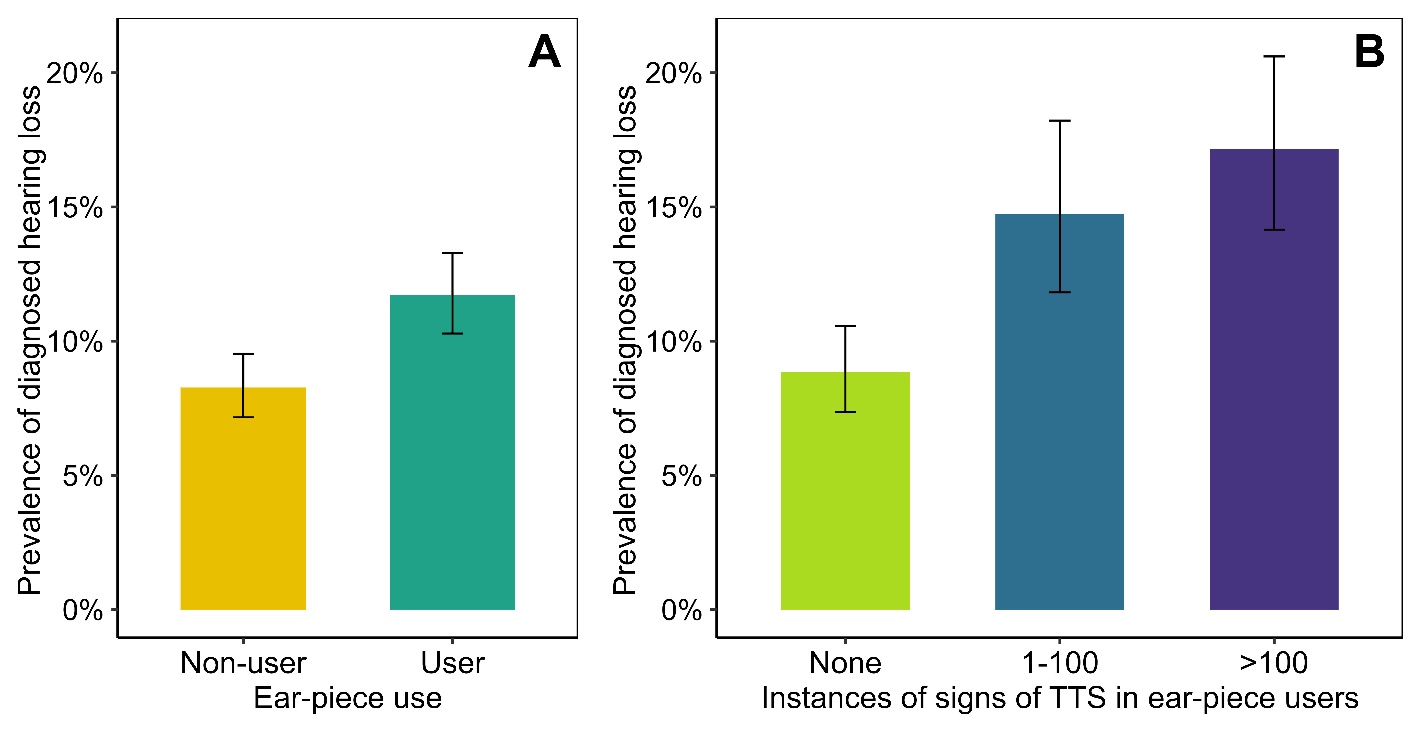


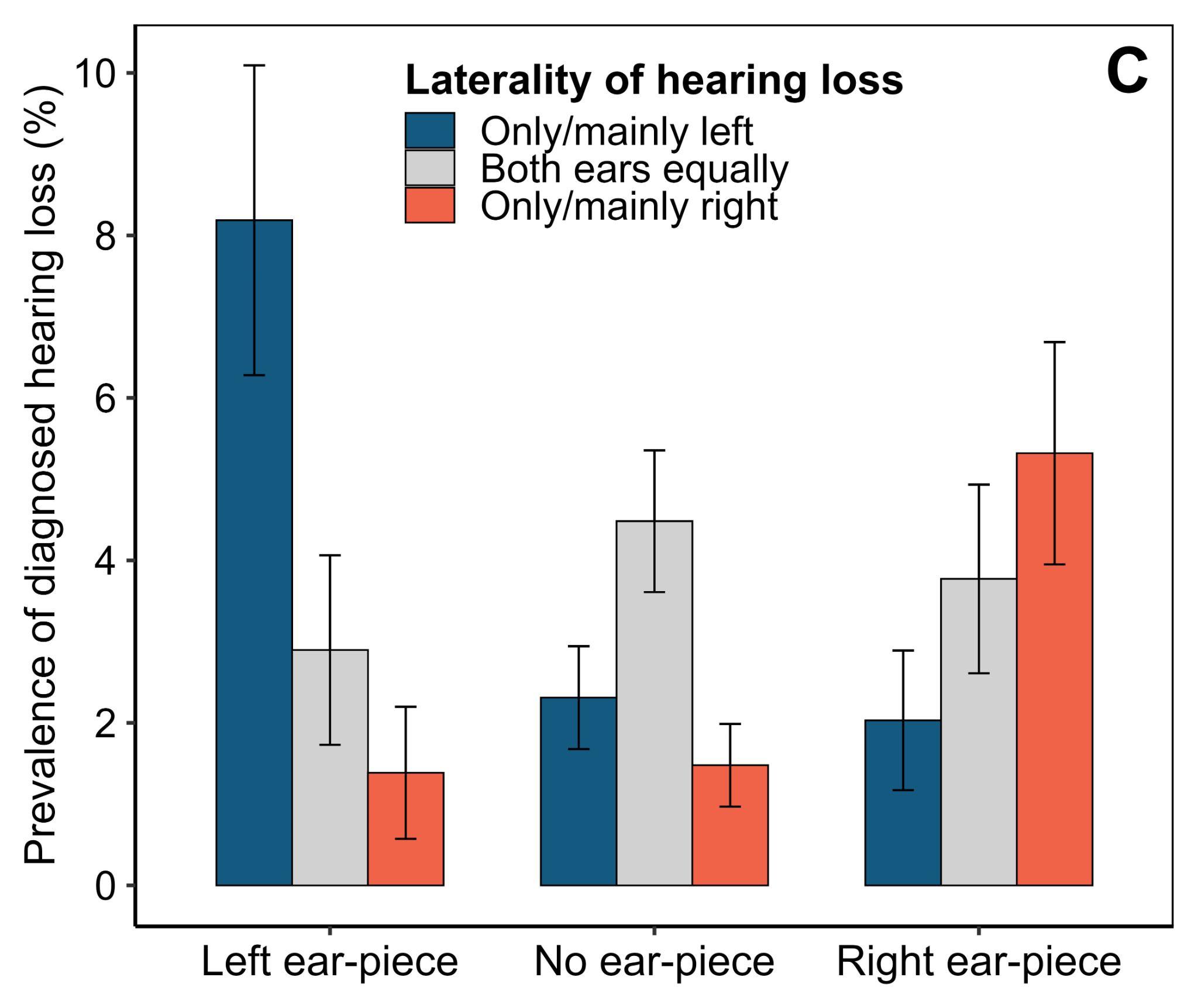


##### Figure SM3. A: Prevalence of diagnosed hearing loss for ear-piece users vs non-users. B: Prevalence of diagnosed hearing loss for three groups of ear-piece users: those who had not experienced signs of ear-piece-induced TTS, those who reported experiencing them on 1-100 occasions, and those who reported experiencing them on >100 occasions. C: Prevalence of diagnosed hearing loss categorised based on laterality (affecting both ears equally, only/mainly the right ear, or only/mainly the left ear) for three exposure groups (non-ear-piece users, right-sided ear-piece users, and left-sided ear-piece users). In all three plots, error bars represent unadjusted 95% confidence intervals, calculated using a binomial approximation based on the observed proportions, and not adjusted associations obtained from regression analysis.

## Tinnitus

### To supplement the RQ4 analysis reported in the main paper, a secondary analysis was performed, whose explanatory variable was total energy of noise exposure (*DeviceNoise*) rather than the binary measure of ear-piece use (*DeviceUse*). The median value for *DeviceNoise* was 18.4 units of noise exposure for participants with tinnitus and 13.5 units for participants without tinnitus. The formal analysis used a multiple logistic regression model specification nearly identical to that of RQ4, substituting *DeviceNoise* for *DeviceUse*. The results indicated that the risk of prolonged spontaneous tinnitus (*TinLong*) was positively associated with total energy of noise exposure (*DeviceNoise*), controlling for age (*p* < 0.00001).

## Demographic factors

A subset of the study RQs (RQ3, RQ4, and RQ5) could conceivably be influenced by age and/or sex. We explored the effects of adding terms representing these demographic factors to the analysis models. Note that effects of ethnicity could not be analysed, due to the low prevalence (~4%) of non-white ethnicities in the study sample.

The primary RQ3 model tested for a relation between ear-piece volume-control setting and rate of occurrence of TTS. Versions of this model with added demographic covariates indicated no statistically significant effect of age (*p* = 0.12) or sex (*p* = 0.42) on the outcome; for each, the effect of volume-control setting remained robust (*p* < 0.00001).

The primary RQ4 model tested for a relation between ear-piece use and risk of prolonged spontaneous tinnitus, controlling for age. Age was associated with increased risk of tinnitus (OR = 1.04 per one-year increase in age, 95% CI 1.02 to 1.05, *p* < 0.00001). Potential effects of sex were also of interest, since ear-piece use was more prevalent for males (52.5%) than for females (33.5%) and tinnitus was also more prevalent for males (16.6%) than for females (14.9%). Hence, an exploratory version of the RQ4 model was constructed, with sex added as a covariate; this revealed no significant effect of sex on tinnitus (*p* = 0.69) and the effect of ear-piece use remained robust (*p* < 0.00001).

The primary RQ5 model tested for a relation between instances of signs of TTS and risk of prolonged spontaneous tinnitus, controlling for age. Consistent with the RQ4 analysis above, age was associated with increased risk of tinnitus (OR = 1.04 per one-year increase in age, 95% CI 1.02 to 1.05, *p* < 0.00001). Sex did not differ substantially across the three TTS groups: the group that reported no signs of TTS was 75.0% male, the group that reported “1-100” instances was 76.3% male, and the group that reported “>100” instances was 73.6% male. Adding sex to the RQ5 model revealed no significant effect on tinnitus (*p* = 0.63) and the primary association between signs of TTS and tinnitus risk remained robust (*p* < 0.00001).
